# ‘Classical’ but not ‘other’ mutations of EGFR kinase domain are associated with clinical outcome in gefitinib-treated patients with non-small cell lung cancer

**DOI:** 10.1038/sj.bjc.6604068

**Published:** 2007-11-13

**Authors:** A G Pallis, A Voutsina, Ar Kalikaki, J Souglakos, E Briasoulis, S Murray, A Koutsopoulos, M Tripaki, E Stathopoulos, D Mavroudis, V Georgoulias

**Affiliations:** 1 Department of Medical Oncology, University General Hospital of Heraklion, Heraklion, Greece; 2 Laboratory of Tumor Cell Biology, School of Medicine, University of Crete, Heraklion, Greece; 3 Department of Medical Oncology, University General Hospital of Ioannina, Ioannina, Greece; 4 Department of Molecular Biology and Genetics, Metropolitan Hospital, Athens, Greece; 5 Department of Pathology, University General Hospital of Heraklion, Heraklion, Greece

**Keywords:** NSCLC, EGFR, gefitinib, EGFR mutations

## Abstract

‘Classical’ mutations in the EGFR tyrosine kinase domain (exons 18, 19 and 21) have been associated with sensitivity to tyrosine kinase inhibitors (TKIs) in patients with NSCLC. The aim of the current study was to evaluate whether other than the classical G719X, DEL19 and L858R mutations of EGFR confer sensitivity to TKIs. Genomic DNA was extracted from microdissected formalin-fixed paraffin-embedded tumour tissue from 86 patients treated with gefitinib. Exons 18, 19 and 21 were amplified and subjected to direct sequencing. Eleven (13%) patients harboured the classical exon's 18, 19 and 21 mutations, while 14 (16%) had ‘other’ variants. There was a significantly higher percentage of ‘never-smoker’ patients with ‘classical’ EGFR mutations (*P*=0.002). Among patients with ‘classical’ mutations 3 patients achieved PR and 7 SD, while in the ‘other’ mutations group 10 patients had SD as best response. In the wild-type group, there were 3 patients with PR and 25 with SD. Median TTP was 16, 64 (*P*=0.002) and 21 weeks and median survival was 36, 78 and 67 weeks for patients with wild-type, ‘classical’ and ‘other’ EGFR mutations, respectively. The clinical relevance of ‘other’ EGFR mutation variants remains uncertain and requires further assessment in a prospective study.

Despite the use of newer chemotherapeutic agents, survival of patients with advanced/metastatic NSCLC after first- or second-line chemotherapy seems to have reached a plateau (Carney, 2002). Further improvement in treatment is likely to require integration of novel, molecular agents such as EGFR inhibitors. The BR.21 placebo-controlled trial of erlotinib demonstrated that therapy with this EGFR tyrosine kinase inhibitor (TKI) was associated with statistically significant and clinically relevant differences in terms of overall and progression-free survival compared to the placebo (Shepherd *et al*, 2005). Conversely, in a similar study with gefitinib, (ISEL trial), a survival benefit was not demonstrated (Thatcher *et al*, 2005). Several clinical factors have been correlated with response to TKIs, including never-smoking status, female sex, Asian ethnicity and adenocarcinoma histology (Fukuoka *et al*, 2003; Kris *et al*, 2003; Miller *et al*, 2004; Shepherd *et al*, 2005). In addition, several groups have shown that somatic mutations in the tyrosine kinase domain of the EGFR gene, in the exons 18 (G719A/C/S), 19 (DEL19, in-frame deletions which eliminate four to six amino acids just downstream of a critical lysine residue at position 745) and 21 (L858R) (commonly reported as ‘classical’ mutations), were significantly correlated with clinical response to gefitinib therapy (Lynch *et al*, 2004; Paez *et al*, 2004; Eberhard *et al*, 2005; Tsao *et al*, 2005). Patients with ‘classical’ EGFR mutations exhibit objective responses ranging from 75 to 95% (Huang *et al*, 2004; Han *et al*, 2005; Mitsudomi *et al*, 2005; Taron *et al*, 2005; Tokumo *et al*, 2005; Inoue *et al*, 2006; Yoshida *et al*, 2007). Furthermore, recently it was reported that polysomy or amplification of the EGFR gene but not EGFR mutational status was associated with clinical outcome of patients treated with erlotinib (Hirsch *et al*, 2005; Tsao *et al*, 2005; Dziadziuszko *et al*, 2006).

Although classical EGFR mutations are present in most cases of NSCLC responding to TKIs therapy, approximately 10–20% of patients who do show a clinical response to gefitinib do not have these EGFR mutations, indicating that other, than these classical mutations, may confer sensitivity to TKIs, or that EGFR mutations are not the sole determinants of TKI response (Sharma *et al*, 2007). Since the original reports by Paez *et al* (2004) and Lynch *et al* (2004) several ‘other’ mutations of EGFR gene have been reported (Riely *et al*, 2006b). During sequence analysis performed by our group, several mutations of EGFR's exons 18, 19 and 21, other than the classical ones, which will be collectively reported in the text as ‘other’, have been identified; however, their biological as well as their clinical relevance is still unclear (Marchetti *et al*, 2005). The aim of the present study was to determine whether the presence of these ‘other’ variants is correlated with the clinical outcome of patients treated with gefitinib.

In the present study, the clinical outcome of NSCLC patients treated with gefitinib in the context of an expanded access program (EAP) was analysed according to the presence of classical or ‘other’ variants of EGFR mutations.

## PATIENTS AND METHODS

### Patient selection

Patients with histologically documented NSCLCs were included in this retrospective analysis. Additional inclusion criteria were as follows: a gefitinib treatment of at least 4 weeks (to evaluate efficacy after adequate exposure to the drug); complete information regarding tumour size, tumour location, extent of disease and prior treatments for NSCLC; bi-dimensionally measurable disease with imaging assessment performed at least 3 weeks before starting gefitinib therapy and at least one subsequent imaging assessment; tumour tissue was required for the assessment of EGFR mutations.

### Expanded access program

Gefitinib EAP, was a non-randomised, open-label compassionate use program, which enrolled patients with advanced/metastatic NSCLC. The EAP protocol and informed consent forms were approved by the Scientific and Ethics Committees of the participating Institutions. Patient enrollment began on July 2001 and was closed on April 2006. Eligible patients with NSCLCs (aged⩾18 years) were those who either (i) had disease progression with standard systemic chemotherapy or radiation therapy, or (ii) were ineligible for chemotherapy or radiation therapy, finally.

### Treatment

Gefitinib was administered orally, as a once daily dose of 250 mg. Treatment was continued until disease progression, the appearance of unacceptable toxicity or patient's withdrawal of consent. Objective tumour responses were evaluated according to WHO response criteria (Miller *et al*, 1981).

### Immunohistochemistry

Representative, tumour sections from formalin-fixed paraffin-embedded (FFPE) tumour samples were stained immunohistochemically, as described previously (Koutsopoulos *et al*, 2007), using the mouse monoclonal anti-EGFR antibody (clone H11, code M3563, DakoCytomation, Glostrup, Denmark), in dilution 1/50 and incubation time 1 h at room temperature. For the detection of antigen–antibody reaction the Ultra-Vision detection system AP Polymer kit (catalogue no. TL-125-AL, Lab Vision, Cheshire, UK), was used according to the manufacturer's instructions. Immunoreaction was considered as weakly positive (2+) when more than 10% of the tumour cells showed weak to moderate complete membrane staining or as strongly positive (3+) when a strong complete membrane staining was observed in more than 10% of the tumour cells. All other staining patterns were interpreted as negative (0 or 1+) (Koutsopoulos *et al*, 2007).

### DNA extraction and mutation analysis

All tumour samples were FFPE tissues. Representative sections from tissue used for DNA extraction were stained with H&E and subjected to histopathologic examination. Subsequently, tissue samples were macrodissected or microdissected (piezo power Eppendorf Microdissector, Germany) to ensure that specimens contained at least 80% tumour cells. Approximately 5–10 *μ*m sections or 30–100 000 cells were collected from normal (when available) and tumour samples and placed in 2% SDS/proteinase K (10 mg ml^−1^) at 56°C overnight. DNA was extracted from the FFPE tissue using the MasterPure Complete DNA/RNA Purification kit (EPICENTRE; Biotechnologies, Madison, USA) according to the manufacturer's instructions. Exons 18, 19 and 21 were amplified and subjected to direct sequencing. The PCR primers were as follows:

155273L23(18ex) 5′-TCCCAAACACTCAGTGAAACAAA-3′, 155348L22(18ex) 5′-TGGTCTCACAGGACCACTGATT-3′, 154838U22(18) 5′-TCAGAGCCTGTGTTTCTACCAA-3′, 154899U20(18) 5′-TCCAAATGAGCTGGCAAGTG-3′, 55634U24(19ex) 5′-AAATAATCAGTGTGATTCGTGGAG-3′, 156027L20(19) 5′-TGTGGAGATGAGCAGGGTCT-3′, 156107L22(19ex) 5′-GAGGCCAGTGCTGTCTCTAAGG-3′, 155750U20(19) 5′-GTGCATCGCTGGTAACATCC-3′, 173160L22(21Ex) 5′-CAGCTCTGGCTCACACTACCAG-3′, 173076L19(21) 5′-CATCCTCCCCTGCATGTGT-3′, 172656U22(21Ex) 5′-GCAGCGGGTTACATCTTCTTTC-3′, 172747U19(21) 5′-GCTCAGAGCCTGGCATGAA-3′. The first PCR was carried out in total volume of 10 *μ*l containing 1/10 of the extracted genomic DNA using 1 U of Platinum Taq DNA polymerase (Invitrogen Corporation, Carlsbad, CA, USA). The initial denaturing step was 94°C for 15 min, followed by 35 cycles of denaturing step at 94°C for 20 s, annealing step at 60°C for 30 s and extension step at 72°C for 1 min, ending with a final extension step at 72°C for 7 min. Nested PCR was carried in a total volume of 20 *μ*l and the conditions were identical to the first PCR. Cycle sequencing reactions were performed using the nested PCR primers and the ABI BigDye Terminator kit (v3.1, Applied Biosystems, Foster City, CA, USA) and electrophoresed on an ABI3100 genetic analyzer (Applied Biosystems). Sequence variants were determined using the Seqscape software (Applied Biosystems) and confirmed by an independent PCR amplification and sequencing in both directions.

### Statistical analysis

Descriptive statistics for the patient group are reported as median and range. Statistical comparisons between group rates (proportions) were assessed by Pearson's *χ*^2^-test or Fisher's test where appropriate (Altman, 1991). As this was a compassionate use program, the primary outcome variables were safety, survival and assessment of response. Overall survival (OS) was measured from entry into the study until death; 1-year survival was estimated using the Kaplan–Meier method (Collet, 1994).

## RESULTS

### Patients' demographics

Eighty-six patients with a median age of 61 years (range=35–82) were enrolled in the present analysis. Most of the patients (75%) were male, 53 (61.6%) had an adenocarcinoma histology and 61 (79%) had stage IV disease. A total of 28 (33%) patients were never-smokers. All patients had received a platinum-based chemotherapy, except two patients with severe heart failure, which precluded cisplatin administration. A total of 66 patients had gefitinib as third-line treatment; 14 patients (21%) received taxane-based second-line therapy, 21 (32%) a platinum-based therapy while the remaining 31 (47%) received a gemcitabine or irinotecan-based regimen as second-line treatment. Patients' characteristics are presented in Table 1. EGFR membrane expression was assessed by immunohistochemistry in all tumour specimens; A total of 42 (49%) of these specimens were considered as positive (2+ or 3+) for EGFR expression.

### Mutational analysis

EGFR mutation detection was performed by sequencing exons 18, 19 and 21 in tumours of all patients (*n*=86) and matched-normal tissue (*n*=22) or blood (*n*=3) of patients carrying EGFR mutations.

According to the mutational status, three groups of patients were identified as follows: (i) the ‘wild-type’ group (*n*=61 patients; 71%) with no detectable mutations; (ii) ‘classical’ mutations group (*n*=11 patients, 13%; 6 of these patients harboured the classical exon 19 deletion, one the G719D, one the E746V and three the exon 21 L858R point mutation) and (iii) the ‘other’ mutations group (*n*=14 patients, 16%). EGFR mutational status is presented in Table 2. None of the reported EGFR mutations was found in matched-normal tissues suggesting their somatic origin and eliminating the possibility to be single-nucleotide polymorphisms. A total of 8 (57%) cases had ‘other’ mutations that have been previously reported and 6 cases had novel EGFR mutations.

A total of 1 (1.1%) patient (no. 13) had two ‘other’ mutations, while 3 (3.4%) patients (nos. 9, 11 and 18), who were included in the ‘classical mutations’ group, had both the exon 21 L858R mutation and an ‘other’ mutation (Table 3). The incidence of ‘classical’ mutations was gender – (9.4% for males *vs* 22.7% for females; *P*=0.139) and histology – (11.8% in adenocarcinomas *vs* 14.3% in non-adenocarcinomas; *P*=0.752) independent, while it was significantly higher in ‘never-smoker’ patients compared to ‘smokers’ (32 *vs* 2.1%, respectively; *P*=0.001). Similarly, regarding the ‘other’ variants, there was no difference in their incidence according to sex (male *vs* female: 18.8 *vs* 13.6%, *P*=0.750), histology (adenocarcinoma *vs* other: 15.7 *vs* 20.0%, *P*=0.773) and smoking habits (smoker *vs* never-smoker: 20.8 *vs* 8.0%, *P*=0.200). Finally, no correlation was observed between EGFR expression as assessed by IHC and presence of ‘classical’ mutations (*P*=0.732); conversely, the detection of ‘other’ variants was more frequent in patients with tumours, which did not express EGFR by IHC (28.9 *vs* 8.3%, *P*=0.036).

### Treatment

The median duration of gefitinib administration was 17 weeks (range=4–140). The reason for treatment discontinuation was disease progression in all but six (6.9%) patients (drug-related toxicity (*n*=3 patients) and personal reasons non-related to treatment or the disease (*n*=3 patients)). There was no clear association between treatment duration and any of the following: PS (*P*=0.262), histology (*P*=0.751), disease stage (*P*=0.103), smoking status (*P*=0.950), sex (*P*=0.663) and skin rash (*P*=0.357) and EGFR expression by IHC (*P*=0.254). However, patients belonging to the group with ‘classical’ mutations had significantly longer treatment duration when compared with patients of the ‘wild-type’ group (67 weeks *vs* 17 weeks, respectively; *P*=0.018), while there was a trend towards longer treatment duration when compared with patients belonging to group of ‘other’ mutations (67 *vs* 21 weeks, respectively; *P*=0.069). On the contrary, there was no significantly different treatment duration between patients belonging to the ‘other’ mutations and those of the ‘wild-type’ group (21 and 17 weeks, respectively; *P*=0.141).

### Response to treatment

No patient achieved a complete response, while six (7.0%) experienced a partial response (PR) (overall response rate=7.0%; 95% CI=1.59–12.36) and 40 (46.5%) stable disease (SD); the disease control rate (DCR; PR+SD) was 53.5% (95% CI=42.95–64.03%). Additionally, progressive disease (PD) was observed in 40 (46.5%) patients. Disease control rate was significantly higher for the following: (i) patients with PS 0–1 as compared with those with PS⩾2 (67 and 22%, respectively; *P*<0.001); (ii) women as compared to men (77.3 and 42.9%, respectively; *P*=0.007); (iii) never-smokers *vs* smokers (84 and 34%, respectively; *P*<0.001) and (iv) patients who developed skin rash as compared with those who did not (85.2 and 35.7%, respectively; *P*=0.003). In addition, there was a trend towards a higher DCR in patients with adenocarcinoma compared with those bearing other histologic types (54.9 and 47.1%, respectively; *P*=0.057). Regarding patients achieving SD, significant association was observed for those with a PS of 0–1 (*P*=0.003), never-smoker status (*P*=0.001) and development of skin rash (*P*=0.003); conversely, there was no association between DCR and sex (*P*=0.053) or histology (*P*=0.355) (Table 4).

The DCR was significantly higher in patients of the ‘classical’ mutations than in patients of the ‘wild-type’ (90.9 and 43.3%, respectively; *P*=0.006) group; conversely, there was no significant difference between the DCR observed in patients of the ‘classical’ mutations group and that of patients of the ‘other mutations’ group (90.9 and 57.1%, respectively; *P*=0.090) or of patients of the ‘other mutations’ group and those of the ‘wild-type’ group (57.1 and 43.3%, respectively; *P*=0.386). All patients (six out of six) with exon 19 deletion achieved disease control (two patients achieved PR and four SD); in addition, two out of three patients with exon 21 L858R mutation experienced disease control (one patient with PR and one with SD) while both patients with the G719D and E746V point mutations achieved SD. In the multivariate analysis, the presence of mutations (either ‘classical’, or ‘other’, as well as all patients with mutations) did not emerge as a significant factor associated with DCR or SD.

### Time to tumour progression

**Figure 1:**
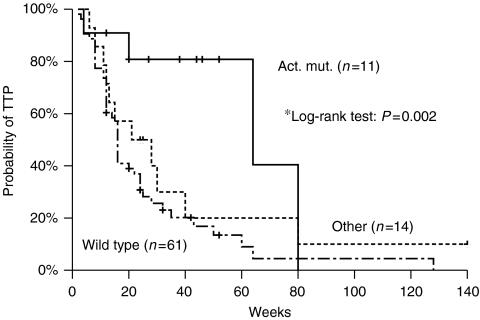
Kaplan–Meier curve of time to tumour progression (TTP) of wild-type EGFR patients group, ‘classical’ and ‘other’ mutations group. ^*^P-value between ‘classical’ and wild type.

The median follow-up period was 109 weeks and the median time to tumour progression (TTP) 20 weeks (range=4–140). A total of 23 (36%) patients had a TTP>24 weeks and 7 (10.9%) >52 weeks (Table 5). There was no difference in TTP according to sex (*P*=0.468), histology (*P*=0.676), EGFR positivity (by IHC) (*P*=0.267) and PS (*P*=0.437); conversely, patients who had never-smoked and patients who developed skin rash had significantly higher TTP (*P*=0.003 and *P*=0.006, respectively). Time to tumour progression in the ‘classical’ mutations group was significantly longer than in the ‘wild-type’ group (64 *vs* 16 weeks; *P*=0.002, Figure 1). On the contrary, there was no difference in TTP between ‘other’ mutations group and ‘wild-type’ group (21 *vs* 16 weeks; *P*=0.363) while there was a trend towards a significant difference in TTP between ‘other’ mutation group and ‘classical’ mutations group (21 *vs* 64 weeks; *P*=0.069). Although patients with DEL19 mutation had numerically higher TTP, when compared with L858R mutation patients, this difference failed to reach statistical significance (80 *vs* 64 weeks, *P*=0.786).

### Survival

**Figure 2:**
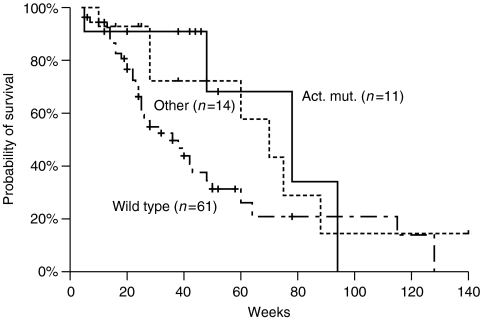
Kaplan–Meier survival curve of wild-type EGFR patients group, ‘classical’ and ‘other’ mutations group.

**Figure 3:**
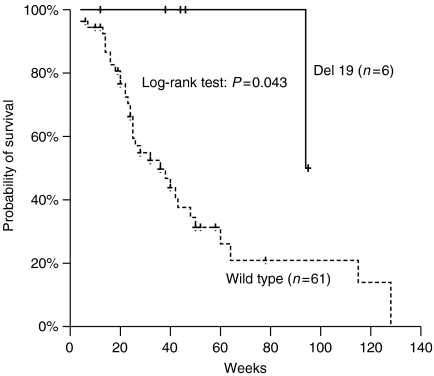
Kaplan–Meier survival curve of patients with the ‘classical’ DEL19 mutation and wild-type EGFR.

Median OS was 48 weeks (range=4–140). None of the following factors had a significant impact on OS: PS (*P*=0.403), histology (*P*=0.198), smoking (*P*=0.242), sex (*P*=0.475), skin rash (*P*=0.182) and EFGR IHC expression (*P*=0.637). Median OS was significantly longer in patients with the DEL19 mutation (not reached) compared to ‘wild-type’ patients (36 weeks; *P*=0.043, Figure 2); however, when all patients with ‘classical’ mutations were included in the analysis, this difference was lost (78 *vs* 36 weeks; *P*=0.052, Figure 3); there was no statistically significant difference regarding OS between patients with DEL19 and L858R mutation (not reached *vs* 78 weeks, *P*=0.896). Similarly, patients of the ‘other’ mutation group had a median survival of 67 weeks, which was not different when compared with ‘wild-type’ group (*P*=0.094) or ‘classical’ mutations group (*P*=0.491). Efficacy results according to mutational status are presented in Table 6.

## DISCUSSION

The incidence of the classical somatic mutations has been reported to range between 3 and 13% in Caucasian populations and between 30 and 40% in populations of Asian descent (Shigematsu *et al*, 2005; Calvo and Baselga, 2006). In a Greek study (Murray *et al*, 2006), somatic mutations of EGFR were reported in 15% of a group of 60 patients with NSCLC, while Dova *et al* (2007) reported two mutations in 50 Greek patients with cancer of unknown primary. In our series, mutational analysis revealed the presence of ‘classical’ mutations in 11 (13%) patients which is in agreement with other reports (Murray *et al*, 2006; Dova *et al*, 2007), while 14 (16%) patients had ‘other’ mutations. A similar high incidence of ‘other’ EGFR mutations has been reported by Tsao *et al* (2005) who have also used microdissected FFPE tumour samples. Microdissection of tumour samples with a low percentage and/or uneven distribution of cancer cells allows the detection of mutations with higher sensitivity; this could be the reason for the high incidence of ‘other’ mutations observed in our study. On the other hand, the low-DNA template input in PCR could generate false mutations. However, in our case the latter is unlikely, given that the sequence analysis of FFPE normal tissue specimens of 22 patients with EGFR mutations revealed no mutations (data not shown).

The presence of ‘other’ mutations in our study was not correlated with sensitivity to gefitinib. Indeed, patients whose tumours harbour ‘other’ variants of EGFR mutations had a higher, but not statistically significant, DCR when compared with patients bearing wild-type EGFR. However, this observation should be considered with caution given the small number of patients analysed. On the other hand, tumour growth control was significantly higher (*P*=0.013) in patients who presented the classical EGFR mutations compared to that of patients with wild-type EGFR, as already has been reported (Argiris *et al*, 2003; Miller *et al*, 2004; Han *et al*, 2005; Kim *et al*, 2005; Thatcher *et al*, 2005; Tsao *et al*, 2005). Similarly, although patients with ‘other’ EGFR mutations had a numerically longer survival as compared with patients of the ‘wild-type’ group, this difference failed to reach statistical significance. This observation may suggest that, collectively, ‘other’ EGFR mutation variants *per se* could not be considered as predictors of clinical outcome.

Furthermore, there was a trend towards higher survival for patients with classical mutations, when compared with ‘wild-type’ patients, but this difference failed to reach statistical significance. A possible reason for this observation is the small number of patients studied. This observation is similar with that reported in the BR.21 study (Tsao *et al*, 2005) but it is in conflict with that reported by other studies (Hirsch *et al*, 2005; Takano *et al*, 2005). However, patients with exon 19 deletions had significantly longer survival than patients of the ‘wild-type’ group; it should be interesting to mention that patients with EGFR exon 19 deletions are reported to have a longer survival than patients with EGFR L858R point mutations when treated with TKIs (Mitsudomi *et al*, 2005; Jackman *et al*, 2006; Riely *et al*, 2006a).

In the original reports by Lynch *et al* (2004) and Paez *et al* (2004), only one mutation per tumour was detected. However, subsequent studies demonstrated the presence of more than one mutation per tumour sample (Huang *et al*, 2004; Pao *et al*, 2004; Taron *et al*, 2005; Murray *et al*, 2006). In the present study, four patients had both a ‘classical’ and ‘other’ mutation variants; three of them achieved SD (Table 3). However, definitive conclusions cannot be drawn, given the small number of patients and the little amount of data presented in the literature.

In summary, our findings demonstrate that classical but not ‘other’ mutation variants of EGFR gene are associated with a higher DCR and better TTP; however, EGFR mutational status should not be considered as the only predictor of response to TKIs since disease control with gefitinib has also been observed in patients without EGFR mutations. Patients harbouring ‘other’ EGFR mutation variants have a clinical comportment comparable with that of patients with wild-type EGFR; although this observation strongly suggests that these mutations *per se* could not confer sensitivity to TKTs, we cannot exclude that some of these mutations could be of clinical relevance. Therefore, it should be of importance to establish a large international database of ‘other’ EGFR mutation variants, to better understand and evaluate their clinical relevance.

## Figures and Tables

**Table 1:** Patients' characteristics

	***n***	**%**
*Age*		
Median	61	
(minimum–maximum)	35–82	
		
*Sex*		
Male	64	75
Female	22	26
		
*Histology*		
Adenocarcinoma	47 (53)	55 (61.6)
Squamous cell carcinoma	27 (29)	31 (33.7)
Large cell carcinoma	1	1 (1.1)
Bronchoalveolar	4	5 (4.7)
Other	7	8 (9.3)
		
*PS (WHO)*		
0–1	53	62
⩾2	33	38
		
*Stage*		
IIIB	25	21
IV	61	79
		
*EGFR expression (IHC)*		
0–1+	44	51
2–3+	42	49
		
*Line of therapy*		
2nd	20	24
⩾3rd	66	76
		
*Smoking status*		
Smoker	39	45
Ex-smoker	19	22
Never-smoker	28	33
		
*No. of organs involved*		
1	19	22
2	48	56
⩾3	19	22
Median (range)	2 (1–4)	

**Table 2:** Results of EGFR mutational analysis

**Patients no.**	**‘Classical mutations’ group**	**‘Other mutations’ group**	**Histology**	**Gender**	**Smoking status**	**Response**	
1	DEL 19		Squamous	Male	Never-smoker	SD	
2		Y727H	Squamous	Male	Smoker	SD	Y727C (273T cell line) reported (Stabile *et al*, 2005)
3	DEL 19		Adenocarcinoma	Male	Never-smoker	SD	
4		V843I	Squamous	Male	Ex-smoker	SD	V843I reported (Koyama *et al*, 2006)
5		L747S	Adenocarcinoma	Female	Never-smoker	SD	L747P reported (Sunaga *et al*, 2007)
6	G719D		Adenocarcinoma	Male	Never-smoker	SD	
7	DEL 19		Adenocarcinoma	Female	Never-smoker	PR	
8		P691S	Adenocarcinoma	Male	Ex-smoker	SD	Novel
9	L858R, L861P		Undifferentiated	Male	Smoker	PD	
10		K860E	Squamous	Male	Smoker	PD	Novel
11	L858R, V843I		Adenocarcinoma	Female	Never-smoker	SD	
12		G863S	Adenocarcinoma	Female	Never-smoker	SD	G863D reported (Bell *et al*, 2005; Chou *et al*, 2005; Riely *et al*, 2006b)
13		T847A, G863S	Adenocarcinoma	Male	Smoker	PD	T847I reported (Tsao *et al*, 2005)
14		L692P	Squamous	Male	Smoker	PD	Possible non-somatic
15		L703F	Adenocarcinoma	Female	Smoker	PD	L703V reported (Takano *et al*, 2005; Riely *et al*, 2006b)
16	E746V		Adenocarcinoma	Female	Never-smoker	SD	
17		G729R	Undifferentiated	Male	Smoker	PD	G729E reported (Willmore-Payne *et al*, 2006)
18	L858R, E709K		Squamous	Female	Never-smoker	PR	
19		V726M	Adenocarcinoma	Male	Smoker	SD	Novel
20	DEL19		Adenocarcinoma	Male	Never-smoker	PR	
21	DEL19		Squamous	Male	Never-smoker	SD	
22		G857E	Adenocarcinoma	Male	Smoker	SD	Reported (Hsieh *et al*, 2006)
23		E711K	Squamous	Male	Smoker	PD	Novel
24	DEL19		BAC	Female	Never-smoker	SD	
25		G874S	Adenocarcinoma	Female	Smoker	SD	
Total	11	14					

**Table 3:** Clinical characteristics of patients bearing both a classical and an ‘other’ mutation

**Patients no.**	**Sex**	**Histology**	**Smoking status**	**Response to gefitinib**	**Duration**	
9	Male	Undifferentiated	Smoker	PD	—	L858R-L861P
11	Female	Adenocarcinoma	Never-smoker	SD	64 weeks	L858R-V843I
18	Female	Squamous	Never-smoker	PR	12 weeks	L858R-E709K

**Table 4:** Clinical and molecular characteristics of patients achieving disease control

	***n***	**Female sex *n* (%)**	**Adenocarcinoma histology^a^ *n* (%)**	**Never smoker *n* (%)**	**Skin rash development *n* (%)**	**EGFR mutations**
PR	6	4 (67%)	2 (33%)	5 (83)	4 (67%)	*DEL 19: n*=2 (33%) *L858R: n*=1 (17%) *Wild type: n*=3 (50%)
SD	40	14 (35%)	28 (70%)	18 (45%)	19 (47.5%)	*DEL 19: n*=4 (10%) *L858R: n*=1 (2.5%) *Other variant: n*=10 (25%) *Wild type: n*=25 (62.5%)

PR=partial response, SD=stable disease.

a Bronchoalveolar included.

**Table 5:** EGFR mutation status for patients with ⩾24 and ⩾52 weeks TTP

**TTP**	***n***	**Wild type**	**Exon 19 deletion**	**L858R**	**Other variant**
⩾24 weeks	23	11 (48%)	5 (22%)	1 (4%)	6 (26%)
⩾52 weeks	7	1 (14%)	2 (29%)	1 (14%)	3 (43%)

**Table 6:** Efficacy results according to mutational status

	**‘Classical’ mutations**	**‘Other’ mutations**	**Wild type**
DCR	90.9%^*^	57.1%	43.3
TTP	64 weeks^*^	21 weeks	16 weeks
OS	78 weeks^**^	67 weeks	36 weeks

DCR=disease control rate, OS=overall survival; TTP=time to tumour progression.

* *P*<0.05 (*vs* wild type).

** *P*=0.052 (*vs* wild type).

## References

[bib1] Altman GD (1991) Practical Statistics for Medical Research. London, UK: Chapman and Hall

[bib2] Argiris A, Mittal N, Masters G (2003) Gefitinib (ZD1839) as first line, compassionate use therapy in patients with advanced NSCLC. Proc Am Soc Clin Oncol 22; Abstract 2729

[bib3] Bell DW, Lynch TJ, Haserlat SM, Harris PL, Okimoto RA, Brannigan BW, Sgroi DC, Muir B, Riemenschneider MJ, Iacona RB, Krebs AD, Johnson DH, Giaccone G, Herbst RS, Manegold C, Fukuoka M, Kris MG, Baselga J, Ochs JS, Haber DA (2005) Epidermal growth factor receptor mutations and gene amplification in non-small-cell lung cancer: molecular analysis of the IDEAL/INTACT gefitinib trials. J Clin Oncol 23: 8081–8092 16204011 10.1200/JCO.2005.02.7078

[bib4] Calvo E, Baselga J (2006) Ethnic differences in response to epidermal growth factor receptor tyrosine kinase inhibitors. J Clin Oncol 24: 2158–2163 16682734 10.1200/JCO.2006.06.5961

[bib5] Carney DN (2002) Lung cancer – time to move on from chemotherapy. N Engl J Med 346: 126–128 11784881 10.1056/NEJM200201103460211

[bib6] Chou TY, Chiu CH, Li LH, Hsiao CY, Tzen CY, Chang KT, Chen YM, Perng RP, Tsai SF, Tsai CM (2005) Mutation in the tyrosine kinase domain of epidermal growth factor receptor is a predictive and prognostic factor for gefitinib treatment in patients with non-small cell lung cancer. Clin Cancer Res 11: 3750–3757 15897572 10.1158/1078-0432.CCR-04-1981

[bib7] Collet D (1994) Modelling Survival Data in Medical Research 3rd edn, Oxford: Blackwell Scientific

[bib8] Dova L, Pentheroudakis G, Georgiou I, Malamou-Mitsi V, Vartholomatos G, Fountzilas G, Kolaitis N, Kitsiou E, Pavlidis N (2007) Global profiling of EGFR gene mutation, amplification, regulation and tissue protein expression in unknown primary carcinomas: to target or not to target? Clin Exp Metastasis 24: 79–86 17390112 10.1007/s10585-007-9055-0

[bib9] Dziadziuszko R, Witta SE, Cappuzzo F, Park S, Tanaka K, Danenberg PV, Baron AE, Crino L, Franklin WA, Bunn Jr PA, Varella-Garcia M, Danenberg KD, Hirsch FR (2006) Epidermal growth factor receptor messenger RNA expression, gene dosage, and gefitinib sensitivity in non-small cell lung cancer. Clin Cancer Res 12: 3078–3084 16707605 10.1158/1078-0432.CCR-06-0106

[bib10] Eberhard DA, Johnson BE, Amler LC, Goddard AD, Heldens SL, Herbst RS, Ince WL, Janne PA, Januario T, Johnson DH, Klein P, Miller VA, Ostland MA, Ramies DA, Sebisanovic D, Stinson JA, Zhang YR, Seshagiri S, Hillan KJ (2005) Mutations in the epidermal growth factor receptor and in KRAS are predictive and prognostic indicators in patients with non-small-cell lung cancer treated with chemotherapy alone and in combination with erlotinib. J Clin Oncol 23: 5900–5909 16043828 10.1200/JCO.2005.02.857

[bib11] Fukuoka M, Yano S, Giaccone G, Tamura T, Nakagawa K, Douillard JY, Nishiwaki Y, Vansteenkiste J, Kudoh S, Rischin D, Eek R, Horai T, Noda K, Takata I, Smit E, Averbuch S, Macleod A, Feyereislova A, Dong RP, Baselga J (2003) Multi-institutional randomized phase II trial of gefitinib for previously treated patients with advanced non-small-cell lung cancer. J Clin Oncol 21: 2237–2246 12748244 10.1200/JCO.2003.10.038

[bib12] Han SW, Kim TY, Hwang PG, Jeong S, Kim J, Choi IS, Oh DY, Kim JH, Kim DW, Chung DH, Im SA, Kim YT, Lee JS, Heo DS, Bang YJ, Kim NK (2005) Predictive and prognostic impact of epidermal growth factor receptor mutation in non-small-cell lung cancer patients treated with gefitinib. J Clin Oncol 23: 2493–2501 15710947 10.1200/JCO.2005.01.388

[bib13] Hirsch FR, Varella-Garcia M, McCoy J, West H, Xavier AC, Gumerlock P, Bunn Jr PA, Franklin WA, Crowley J, Gandara DR (2005) Increased epidermal growth factor receptor gene copy number detected by fluorescence *in situ* hybridization associates with increased sensitivity to gefitinib in patients with bronchioloalveolar carcinoma subtypes: a Southwest oncology group study. J Clin Oncol 23: 6838–6845 15998906 10.1200/JCO.2005.01.2823

[bib14] Hsieh MH, Fang YF, Chang WC, Kuo HP, Lin SY, Liu HP, Liu CL, Chen HC, Ku YC, Chen YT, Chang YH, Chen YT, Hsi BL, Tsai SF, Huang SF (2006) Complex mutation patterns of epidermal growth factor receptor gene associated with variable responses to gefitinib treatment in patients with non-small cell lung cancer. Lung Cancer 53: 311–322 16870303 10.1016/j.lungcan.2006.06.005

[bib15] Huang SF, Liu HP, Li LH, Ku YC, Fu YN, Tsai HY, Chen YT, Lin YF, Chang WC, Kuo HP, Wu YC, Chen YR, Tsai SF (2004) High frequency of epidermal growth factor receptor mutations with complex patterns in non-small cell lung cancers related to gefitinib responsiveness in Taiwan. Clin Cancer Res 10: 8195–8203 15623594 10.1158/1078-0432.CCR-04-1245

[bib16] Inoue A, Suzuki T, Fukuhara T, Maemondo M, Kimura Y, Morikawa N, Watanabe H, Saijo Y, Nukiwa T (2006) Prospective phase II study of gefitinib for chemotherapy-naive patients with advanced non-small-cell lung cancer with epidermal growth factor receptor gene mutations. J Clin Oncol 24: 3340–3346 16785471 10.1200/JCO.2005.05.4692

[bib17] Jackman DM, Yeap BY, Sequist LV, Lindeman N, Holmes AJ, Joshi VA, Bell DW, Huberman MS, Halmos B, Rabin MS, Haber DA, Lynch TJ, Meyerson M, Johnson BE, Janne PA (2006) Exon 19 deletion mutations of epidermal growth factor receptor are associated with prolonged survival in non-small cell lung cancer patients treated with gefitinib or erlotinib. Clin Cancer Res 12: 3908–3914 16818686 10.1158/1078-0432.CCR-06-0462

[bib18] Kim KS, Jeong JY, Kim YC, Na KJ, Kim YH, Ahn SJ, Baek SM, Park CS, Park CM, Kim YI, Lim SC, Park KO (2005) Predictors of the response to gefitinib in refractory non-small cell lung cancer. Clin Cancer Res 11: 2244–2251 15788673 10.1158/1078-0432.CCR-04-2081

[bib19] Koutsopoulos AV, Mavroudis D, Dambaki KI, Souglakos J, Tzortzaki EG, Drositis J, Delides GS, Georgoulias V, Stathopoulos EN (2007) Simultaneous expression of c-erbB-1, c-erbB-2, c-erbB-3 and c-erbB-4 receptors in non-small-cell lung carcinomas: correlation with clinical outcome. Lung Cancer 57: 193–200 17442448 10.1016/j.lungcan.2007.03.009

[bib20] Koyama N, Jinn Y, Takabe K, Yoshizawa M, Usui Y, Inase N, Miyake S, Yoshizawa Y, Hagiwara K, Kanazawa M (2006) The characterization of gefitinib sensitivity and adverse events in patients with non-small cell lung cancer. Anticancer Res 26: 4519–4525 17201173

[bib21] Kris MG, Natale RB, Herbst RS, Lynch Jr TJ, Prager D, Belani CP, Schiller JH, Kelly K, Spiridonidis H, Sandler A, Albain KS, Cella D, Wolf MK, Averbuch SD, Ochs JJ, Kay AC (2003) Efficacy of gefitinib, an inhibitor of the epidermal growth factor receptor tyrosine kinase, in symptomatic patients with non-small cell lung cancer: a randomized trial. JAMA 290: 2149–2158 14570950 10.1001/jama.290.16.2149

[bib22] Lynch TJ, Bell DW, Sordella R, Gurubhagavatula S, Okimoto RA, Brannigan BW, Harris PL, Haserlat SM, Supko JG, Haluska FG, Louis DN, Christiani DC, Settleman J, Haber DA (2004) Activating mutations in the epidermal growth factor receptor underlying responsiveness of non-small-cell lung cancer to gefitinib. N Engl J Med 350: 2129–2139 15118073 10.1056/NEJMoa040938

[bib23] Marchetti A, Martella C, Felicioni L, Barassi F, Salvatore S, Chella A, Camplese PP, Iarussi T, Mucilli F, Mezzetti A, Cuccurullo F, Sacco R, Buttitta F (2005) EGFR mutations in non-small-cell lung cancer: analysis of a large series of cases and development of a rapid and sensitive method for diagnostic screening with potential implications on pharmacologic treatment. J Clin Oncol 23: 857–865 15681531 10.1200/JCO.2005.08.043

[bib24] Miller AB, Hoogstraten B, Staquet M, Winkler A (1981) Reporting results of cancer treatment. Cancer 47: 207–214 7459811 10.1002/1097-0142(19810101)47:1<207::aid-cncr2820470134>3.0.co;2-6

[bib25] Miller VA, Kris MG, Shah N, Patel J, Azzoli C, Gomez J, Krug LM, Pao W, Rizvi N, Pizzo B, Tyson L, Venkatraman E, Ben-Porat L, Memoli N, Zakowski M, Rusch V, Heelan RT (2004) Bronchioloalveolar pathologic subtype and smoking history predict sensitivity to gefitinib in advanced non-small-cell lung cancer. J Clin Oncol 22: 1103–1109 15020612 10.1200/JCO.2004.08.158

[bib26] Mitsudomi T, Kosaka T, Endoh H, Horio Y, Hida T, Mori S, Hatooka S, Shinoda M, Takahashi T, Yatabe Y (2005) Mutations of the epidermal growth factor receptor gene predict prolonged survival after gefitinib treatment in patients with non-small-cell lung cancer with postoperative recurrence. J Clin Oncol 23: 2513–2520 15738541 10.1200/JCO.2005.00.992

[bib27] Murray S, Timotheadou E, Linardou H, Vrettou AV, Kostopoulos I, Skrickova J, Papakostantinou C, Christodoulou C, Pectasides D, Samantas E, Papakostas P, Skarlos DV, Kosmidis P, Fountzilas G (2006) Mutations of the epidermal growth factor receptor tyrosine kinase domain and associations with clinicopathological features in non-small cell lung cancer patients. Lung Cancer 52: 225–233 16567021 10.1016/j.lungcan.2005.12.015

[bib28] Paez JG, Janne PA, Lee JC, Tracy S, Greulich H, Gabriel S, Herman P, Kaye FJ, Lindeman N, Boggon TJ, Naoki K, Sasaki H, Fujii Y, Eck MJ, Sellers WR, Johnson BE, Meyerson M (2004) EGFR mutations in lung cancer: correlation with clinical response to gefitinib therapy. Science 304: 1497–1500 15118125 10.1126/science.1099314

[bib29] Pao W, Miller V, Zakowski M, Doherty J, Politi K, Sarkaria I, Singh B, Heelan R, Rusch V, Fulton L, Mardis E, Kupfer D, Wilson R, Kris M, Varmus H (2004) EGF receptor gene mutations are common in lung cancers from ‘never smokers’ and are associated with sensitivity of tumors to gefitinib and erlotinib. Proc Natl Acad Sci USA 101: 13306–13311 15329413 10.1073/pnas.0405220101 PMC516528

[bib30] Riely GJ, Pao W, Pham D, Li AR, Rizvi N, Venkatraman ES, Zakowski MF, Kris MG, Ladanyi M, Miller VA (2006a) Clinical course of patients with non-small cell lung cancer and epidermal growth factor receptor exon 19 and exon 21 mutations treated with gefitinib or erlotinib. Clin Cancer Res 12: 839–844 16467097 10.1158/1078-0432.CCR-05-1846

[bib31] Riely GJ, Politi KA, Miller VA, Pao W (2006b) Update on epidermal growth factor receptor mutations in non-small cell lung cancer. Clin Cancer Res 12: 7232–7241 17189394 10.1158/1078-0432.CCR-06-0658

[bib32] Sharma SV, Bell DW, Settleman J, Haber DA (2007) Epidermal growth factor receptor mutations in lung cancer. Nat Rev Cancer 7: 169–181 17318210 10.1038/nrc2088

[bib33] Shepherd FA, Rodrigues PJ, Ciuleanu T, Tan EH, Hirsh V, Thongprasert S, Campos D, Maoleekoonpiroj S, Smylie M, Martins R, van KM, Dediu M, Findlay B, Tu D, Johnston D, Bezjak A, Clark G, Santabarbara P, Seymour L (2005) Erlotinib in previously treated non-small-cell lung cancer. N Engl J Med 353: 123–132 16014882 10.1056/NEJMoa050753

[bib34] Shigematsu H, Lin L, Takahashi T, Nomura M, Suzuki M, Wistuba II, Fong KM, Lee H, Toyooka S, Shimizu N, Fujisawa T, Feng Z, Roth JA, Herz J, Minna JD, Gazdar AF (2005) Clinical and biological features associated with epidermal growth factor receptor gene mutations in lung cancers. J Natl Cancer Inst 97: 339–346 15741570 10.1093/jnci/dji055

[bib35] Stabile LP, Lyker JS, Gubish CT, Zhang W, Grandis JR, Siegfried JM (2005) Combined targeting of the estrogen receptor and the epidermal growth factor receptor in non-small cell lung cancer shows enhanced antiproliferative effects. Cancer Res 65: 1459–1470 15735034 10.1158/0008-5472.CAN-04-1872

[bib36] Sunaga N, Tomizawa Y, Yanagitani N, Iijima H, Kaira K, Shimizu K, Tanaka S, Suga T, Hisada T, Ishizuka T, Saito R, Dobashi K, Mori M (2007) Phase II prospective study of the efficacy of gefitinib for the treatment of stage III/IV non-small cell lung cancer with EGFR mutations, irrespective of previous chemotherapy. Lung Cancer 56: 383–389 17368623 10.1016/j.lungcan.2007.01.025

[bib37] Takano T, Ohe Y, Sakamoto H, Tsuta K, Matsuno Y, Tateishi U, Yamamoto S, Nokihara H, Yamamoto N, Sekine I, Kunitoh H, Shibata T, Sakiyama T, Yoshida T, Tamura T (2005) Epidermal growth factor receptor gene mutations and increased copy numbers predict gefitinib sensitivity in patients with recurrent non-small-cell lung cancer. J Clin Oncol 23: 6829–6837 15998907 10.1200/JCO.2005.01.0793

[bib38] Taron M, Ichinose Y, Rosell R, Mok T, Massuti B, Zamora L, Mate JL, Manegold C, Ono M, Queralt C, Jahan T, Sanchez JJ, Sanchez-Ronco M, Hsue V, Jablons D, Sanchez JM, Moran T (2005) Activating mutations in the tyrosine kinase domain of the epidermal growth factor receptor are associated with improved survival in gefitinib-treated chemorefractory lung adenocarcinomas. Clin Cancer Res 11: 5878–5885 16115929 10.1158/1078-0432.CCR-04-2618

[bib39] Thatcher N, Chang A, Parikh P, Rodrigues PJ, Ciuleanu T, von PJ, Thongprasert S, Tan EH, Pemberton K, Archer V, Carroll K (2005) Gefitinib plus best supportive care in previously treated patients with refractory advanced non-small-cell lung cancer: results from a randomised, placebo-controlled, multicentre study (Iressa survival evaluation in lung cancer). Lancet 366: 1527–1537 16257339 10.1016/S0140-6736(05)67625-8

[bib40] Tokumo M, Toyooka S, Kiura K, Shigematsu H, Tomii K, Aoe M, Ichimura K, Tsuda T, Yano M, Tsukuda K, Tabata M, Ueoka H, Tanimoto M, Date H, Gazdar AF, Shimizu N (2005) The relationship between epidermal growth factor receptor mutations and clinicopathologic features in non-small cell lung cancers. Clin Cancer Res 11: 1167–1173 15709185

[bib41] Tsao MS, Sakurada A, Cutz JC, Zhu CQ, Kamel-Reid S, Squire J, Lorimer I, Zhang T, Liu N, Daneshmand M, Marrano P, da Cunha SG, Lagarde A, Richardson F, Seymour L, Whitehead M, Ding K, Pater J, Shepherd FA (2005) Erlotinib in lung cancer – molecular and clinical predictors of outcome. N Engl J Med 353: 133–144 16014883 10.1056/NEJMoa050736

[bib42] Willmore-Payne C, Holden JA, Layfield LJ (2006) Detection of EGFR- and HER2-activating mutations in squamous cell carcinoma involving the head and neck. Mod Pathol 19: 634–640 16547470 10.1038/modpathol.3800552

[bib43] Yoshida K, Yatabe Y, Park JY, Shimizu J, Horio Y, Matsuo K, Kosaka T, Mitsudomi T, Hida T (2007) Prospective validation for prediction of gefitinib sensitivity by epidermal growth factor receptor gene mutation in patients with non-small cell lung cancer. J Thorac Oncol 2: 22–28 17410005

